# Predicting adverse drug reactions through interpretable deep learning framework

**DOI:** 10.1186/s12859-018-2544-0

**Published:** 2018-12-28

**Authors:** Sanjoy Dey, Heng Luo, Achille Fokoue, Jianying Hu, Ping Zhang

**Affiliations:** 1 Center for Computational Health, IBM T.J. Watson Research Center, 1101 Kitchawan Road, Yorktown Heights, NY USA; 2 Cognitive Computing, IBM T.J. Watson Research Center, 1101 Kitchawan Road, Yorktown Heights, NY USA

**Keywords:** Chemical fingerprint, Adverse drug reaction, Deep learning

## Abstract

**Background:**

Adverse drug reactions (ADRs) are unintended and harmful reactions caused by normal uses of drugs. Predicting and preventing ADRs in the early stage of the drug development pipeline can help to enhance drug safety and reduce financial costs.

**Methods:**

In this paper, we developed machine learning models including a deep learning framework which can simultaneously predict ADRs and identify the molecular substructures associated with those ADRs without defining the substructures *a-priori*.

**Results:**

We evaluated the performance of our model with ten different state-of-the-art fingerprint models and found that neural fingerprints from the deep learning model outperformed all other methods in predicting ADRs. Via feature analysis on drug structures, we identified important molecular substructures that are associated with specific ADRs and assessed their associations via statistical analysis.

**Conclusions:**

The deep learning model with feature analysis, substructure identification, and statistical assessment provides a promising solution for identifying risky components within molecular structures and can potentially help to improve drug safety evaluation.

## Background

According to the definition by the World Health Organization (WHO), an adverse drug reaction (ADR) is generally defined as an unintended and harmful reaction suspected to be caused by a drug taken under normal conditions [1]. It has been recognized that ADRs represent a significant public health problem all over the world. In the United States, it is estimated that over 2 million serious ADRs occur among hospitalized patients, which results in over 100,000 deaths each year [2, 3]. Identifying potential ADRs of drug candidates in the early stage of the drug development pipeline can improve drug safety, reduce risks for the patients and save money for the pharmaceutical companies.

The information available in the early stages of drug development is mainly the chemical structure of the drug candidate. Many existing studies on ADR prediction have been devoted to analyzing the chemical properties of drug molecules. Though the mechanisms of ADRs are complicated and may not be well understood, machine learning techniques are promising solutions to understand and analyze such complicated problems. In general, the basic steps of ADR prediction based on structural information can be broken down into two stages. First, each drug molecule is represented in a suitable feature vector based on its chemical structure. Second, a machine learning algorithm is applied on the resulting feature space to predict ADRs. So far, most of the existing studies focused on the second step, or the method development, to improve the prediction power [4]. However, how to represent the drug molecules by a useful set of features and how to interpret their effects on the final ADR predictions remain relatively less explored. Note that finding the specific substructures of the drug molecule that is related to an ADR can be particularly useful for finding the mechanism of actions of the drug and thus, can be utilized in the early phase of drug design.

In this paper, we aim to identify and summarize the chemical substructures of drug compounds that have significant associations with ADRs using a machine learning approach, which can provide insights about the connection between structural factors and ADRs. In previous studies [4–8], a set of pre-defined structural features, or fingerprints, are derived first, and then a predictive model is built on them. However, such pre-defined chemical fingerprints do not cover all possibilities of chemical substructures and thus may miss important information. Moreover, these chemical fingerprint algorithms are unsupervised in nature, i.e., they are derived from drug molecules irrespective of the ADR prediction applications. Therefore, these fingerprints only contain generic structural information and may not be optimally associated with ADRs. To identify the substructure features that are not defined *a-priori* and to improve the prediction power of ADRs simultaneously, we leveraged a convolutional deep learning framework [9] to integrate the two stages of ADR predictions, feature creation and predictive model development, into a single system to find chemical substructures associated with ADRs. To make the deep learning framework interpretable enough, we used attention mechanism [10] for finding the specific substructures of the drug. Furthermore, we rank the substructure-ADR association results using statistical analysis and found literature evidence to validate the drug-ADR associations. Finally, we group the significant associations to further enhance the interpretation of obtained results.

In brief, the contribution of the paper can be summarized as below:

- We developed a neural fingerprint method in a simultaneous deep learning framework for ADR prediction, so that the label information (drug-ADR association) can be utilized in the feature generation stage of machine learning process.
- We interpreted the deep learning framework using the attention framework and analyzed the features to identify which substructures within the drug molecules are specifically related to a particular ADR. Additionally, we used statistical measurements to evaluate their associations and test whether the substructures can help to predict ADRs in new drugs.
- We compared our neural fingerprint method with ten different types of chemical fingerprints and used them as features in a predictive model to assess their performance in ADR prediction based on a dataset collected from drug labels.
- We also systematically analyze the relationships among the groups of chemical substructures with the groups of related ADRs.

In the following sections, we will describe our method, the results we got, related work, discussion and conclusion.

## Methods

### Overall workflow

**Fig. 1:**
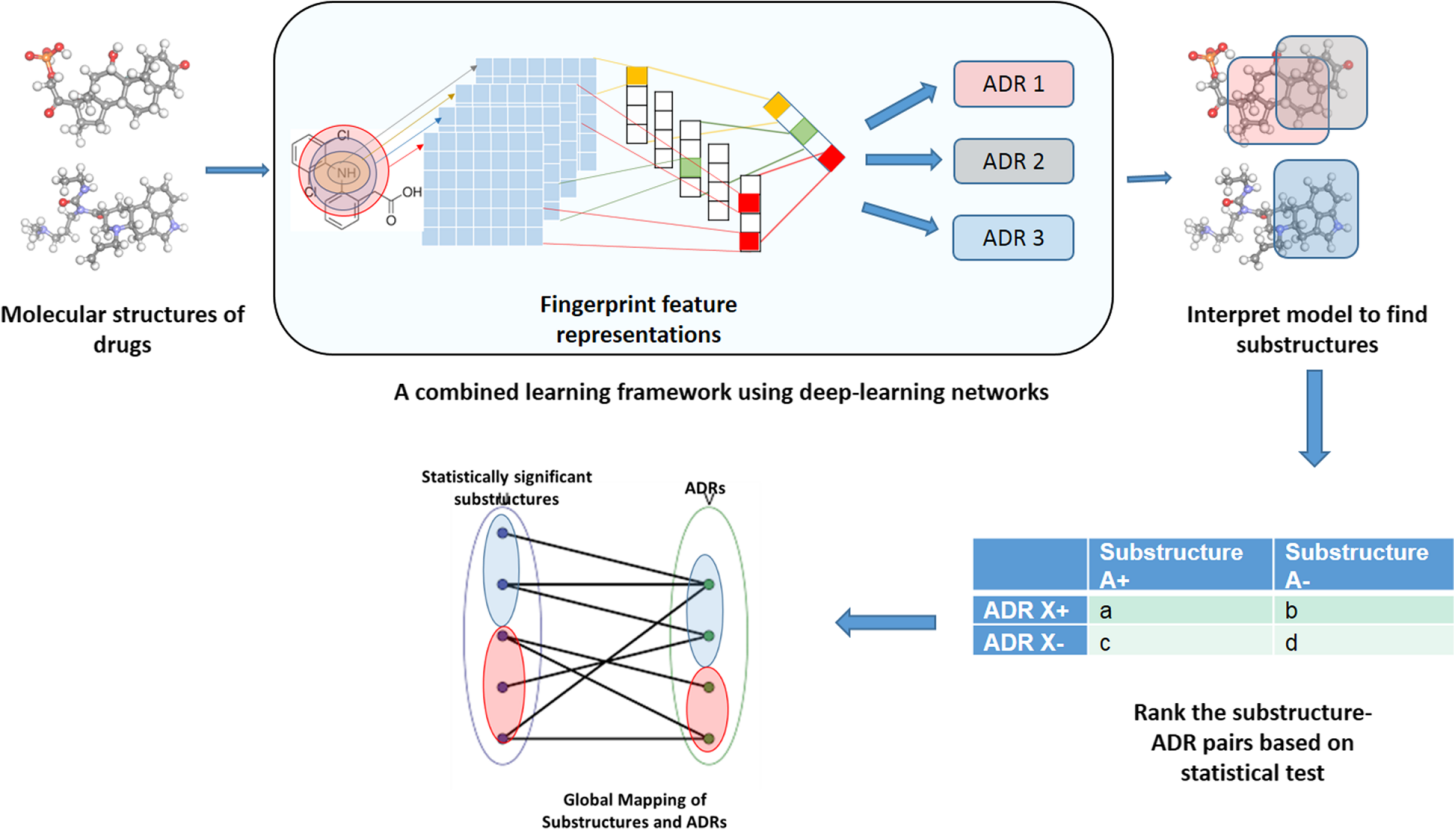
Overall Framework: The general workflow for ADR prediction

The general workflow of this paper is shown in Fig. 1, which consists of the following steps: constructing deep learning fingerprint representations, building predictive models and interpreting those features for characterizing substructures associated with ADRs. Each of these steps are discussed below in detail.

### Constructing chemical fingerprints

**Fig. 2:**
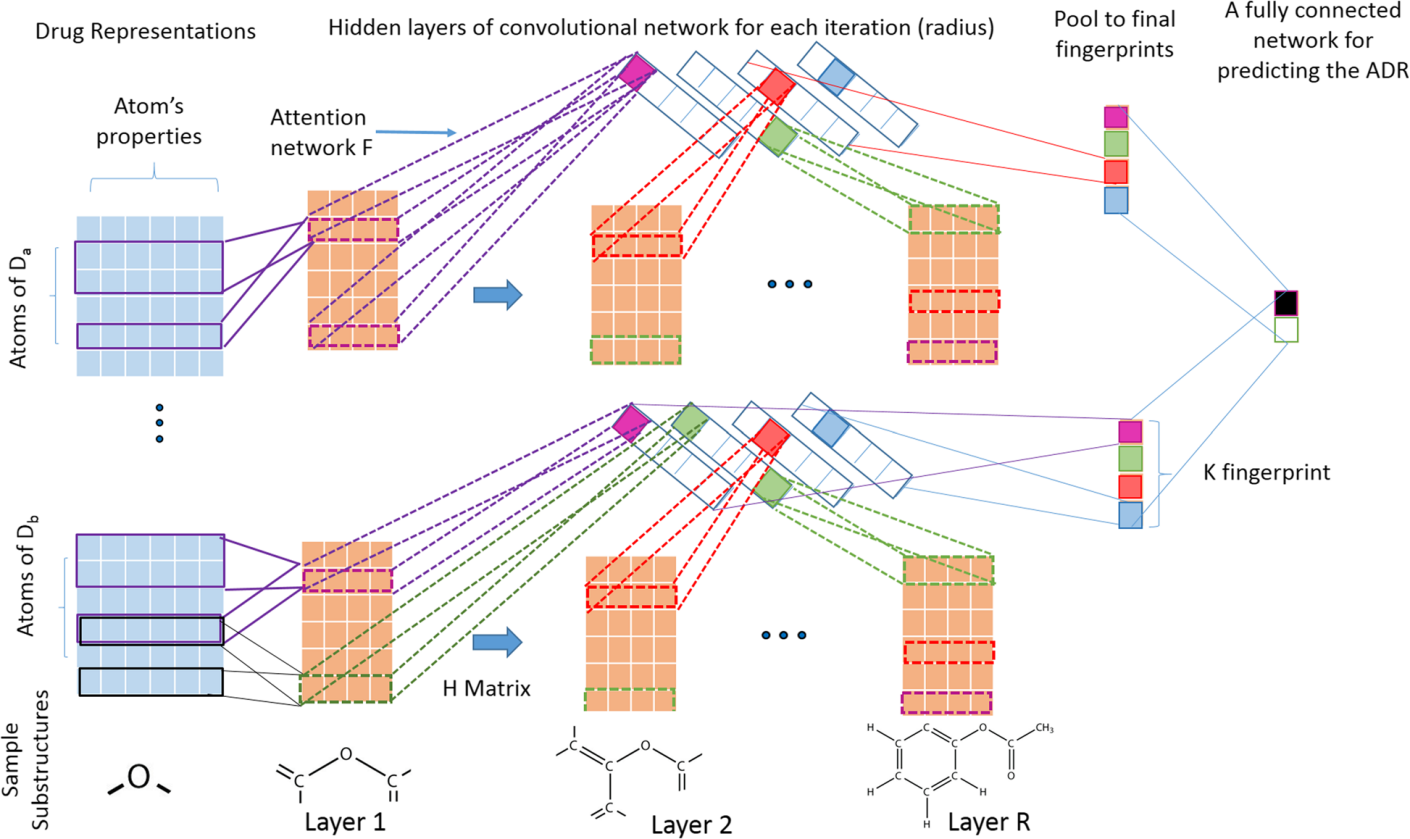
Neural fingerprint method with attention mechanism for predicting an ADR
- **Convolutional feature maps:** The purpose of the convolutional step is to represent a substructure in a particular layer into a condensed feature vector. In every iteration (layer) of the algorithm, each *j*^*th*^ atom of *i*^*th*^ drug in current layer *l*∈{1,2,⋯,*R*} is expanded to include the immediate neighbors of each atom belonging to that substructure. Then, all atomic features and bonding information of the atoms belonging to this expanded substructure at layer *l* are concatenated into a large feature vector noted as $x_{ij}^{l} \in \mathbf {R}^{d_{l}}$ and transformed into new feature vector $x_{ij}^{l+1} \in \mathbf {R}^{d_{l+1}}$ of next layer *l*+1 using convolutional filters. This will represent a substructure denoted by $s_{ij}^{l+1}$ for each atom *j* referred as center and it’s neighbors explored so far in this new layer. Note that each substructure can be obtained by starting the search from multiple atoms belonging to substructures and thus may be obtained from multiple centers. To remove such redundancies we map each substructure $x_{ij}^{l+1}$ into lower dimensions using a single layer of neural network with *d*_*l*_ input nodes and *d*_*l*+1_ output nodes. Therefore, a weight matrix of $\mathbf {H_{l}} \in \mathbf {R}^{d_{l} \times d_{l+1}}$ is defined as a convolutional filter to transform features to next layer as $x_{ij}^{l+1} = f\left (x_{ij}^{l} \mathbf {H_{l}} + b\right)$, where *b*∈**R**. Here, *H*_*l*_ is a hidden-to-hidden filter matrix in each layer *l* and five different types of such filters, $H^{1}_{l} \cdots H^{5}_{l}$ are used for each layer in our case, each corresponds to the number of bonds each atom can have [9]. Also, *f* is a smoothing function to make it insusceptible to minor variations in the substructure. This function is differentiable with respect to the weights **H** and therefore, it can be estimated from the data in an efficient manner.
- **Attention mechanism for representing multiple substructures into fixed sized vectors:** An attention layer network is represented on top of the convolutional features and thus, the network is made interpretable. Specifically, we pooled the similar substructures of the convolution feature maps into a fixed-sized feature vector of size *K* (hyper-parameter representing the length of fingerprint) using another layer of neural network of weights $\phantom {\dot {i}\!}F \in \mathbf {R}^{d_{l} \times K}$. Moreover, a *softmax* function is used on top of this transformation to make it a differentiable index function, since that has been shown to have concise set of fingerprint representations for larger drug molecules [9]. A simple addition function is used to summarize the activation scores of each atom that belongs to a particular molecule in the pooling stage of the convolutional neural network.
- **Final pooling to for getting neural fingerprints:** The previous two steps are iterated for each radius of the molecule upto *R* times, which is the maximum radius of the substructure (another hyper-parameter) using a separate hidden layer to successively explore all possible substructures upto *R* hops. In this paper, we set *R*=4. Finally, the fingerprint vectors obtained from each layer are summarized (pooled) into a final representation by summing up them into a final fixed-length fingerprint representation for each drug.

In this article, we propose a deep learning based framework [11, 12] to learn molecular substructures that are specific to an ADR. The main challenge in representing the molecular graphs of drugs into features is how to represent the varying sizes of each drug molecule into a fixed-size feature representation [13]. To circumvent this problem, we propose a convolutional deep learning based framework similar to [9] so that we can utilize deep learning to simultaneously construct chemical fingerprint features and assess their associations with ADRs. Figure 2 represents the detailed architecture of the framework. Intuitively, the neural fingerprint algorithm explores all possible substructures of the given drug molecules in the training data upto a particular size (often referred as *radius* in literature). Formally, *radius* of a substructure is defined as half of the maximum path length between any two atoms of that substructure. In our neural fingerprint algorithm, we successively explore substructures of all radius upto a user-provided input hyper-parameter *R*. In particular, we design *R* hidden layers in the deep learning framework, each corresponding to a particular radius. Therefore, our framework can search for all possible substructures upto radius *R* by successive increment of the radius of the substructure by one in each layer of neural network. Afterward, the similar structures are summarized into a final feature representations called fingerprint. At each step (radius), we use an additional attention mechanism step to map the contribution of each of the substructures into the final fingerprint. Finally, the fingerprints are assessed in terms of how well they can predict ADRs and then, they are interpreted to infer meaningful associations. In the following, we will describe the details substeps of the framework.

- **Raw features representations:** We represent each drug into a 2D or 3D graphical structure. Then a set of chemical features are extracted for each of the constituent atoms in the drug. In particular, we used a popular chemical fingerprint algorithm, ECFP [14] to derive features such as atom’s element, its degree, the number of attached hydrogen atoms, and the implicit valence, an aromaticity indicator and bond type. We summarize all such information of all atoms of a given drug *i* into a matrix *X*_*i*_ as the initial input to the deep learning framework. More formally, given *N* total number of drugs and *M* total number of ADRs, each drug *i*∈{1,2,⋯,*N*} is represented by a matrix $\mathbf {X_{i}^{l}} \in \mathbf {R}^{n_{i} \times d_{l}}$ at each layer (corresponding to a particular radius) *l*∈{1,2,⋯,*R*}, where *n*_*i*_ represents the number of atoms in drug *i* and *d*_*l*_ represents the total number of features for each atom. Let $x_{ij}^{l} = \left [ x_{ij1}, x_{ij2}, \cdots, x_{{ijd}_{l}} \right ] \in \mathbf {R}^{d_{l}} $ represent the feature vector of *j*^*th*^ atom of *i*^*th*^ drug at layer *l*, where *j*∈{1,⋯,*n*_*i*_}.

### Building predictive models

Once we get a final fingerprint representation for each drug we use a fully connected neural network to assess its ability to predict an ADR, as shown in the last step of Fig. 2. For each ADR, the drugs associated with the ADR were labeled as positives and the rest of the drugs were labeled as negatives. We built a predictive model using L2-norm regularized logistic regression method [15] for each ADR separately using those fingerprints as features. The loss function is described below, where **Z** is the matrix containing all fingerprints for each drug denoted as *z*_*i*_∈**R**^*K*^ and f is a non-linear logistic function along with L2 loss imposed on the weights vector *w*∈**R**^*K*^ defined on top of *z*_*i*_. Furthermore, we also want the neural fingerprint feature representations *z*_*i*_ itself to be sparse to enhance further model interpretability. Optionally, one or more hidden layer can be introduced between the neural fingerprints and the ADR outcome variable to enhance the prediction power.

$$ \mathcal{L}(Z,y,w) \,=\, \sum_{i} Cost\left(y_{i},f\left(z_{i}*w\,+\,b\right)\right) + \lambda_{1} \left\|w\right\|_{2}^{2} + \lambda_{2}\left\| z_{i}\right\|_{2}^{2} $$

Here, *λ*_1_ and *λ*_2_ are hyper-parameter which have to be learnt from the data.

### Interpreting features for substructure analysis

Extraction and interpretation of the important fingerprints of the drugs may help to derive useful knowledge about the ADRs. Given a particular ADR, we back-trace our learnt deep learning framework to find meaningful substructures that are related to that particular ADR. First, we find the top predictive fingerprints (top panels of Fig. 2) for a given ADR based on the learned weights from the final layer of the neural network. Second, for each important fingerprint, we investigate each layer to find the atoms of drugs (*s*_*ij*_) which have the highest activation for that particular fingerprint using the attentiveness weights *F* and *H*. Finally, we reconstruct the substructures by starting from that atom as center and expanding the neighborhood up to that particular layer.

To mathematically evaluate the connections between substructures and ADRs, we calculated a confusion matrix for a given substructure A regarding the specific ADR X from the SIDER database shown in Table 1. In this table, a is the number of drugs that contain substructure A and cause ADR X; b is the number of drugs that do not contain substructure A but trigger ADR X; c is the number of drugs that contain substructure A but have no association towards ADR X; and d is the number of drugs that do not contain substructure A and have no association towards ADR X. We can calculate p value using chi-squared test and odds ratio (OR) to evaluate the association strength between substructure A and ADR X.

**Table 1:** The confusion matrix to evaluate the association between substructure A and ADR X

	Substructure A+	Substructure A-
ADR X+	a	b
ADR X-	c	d

Once we extract all significant substructures that are associated with the ADRs, we aim to further group them into higher levels, since many of the ADRs are inherently related. For example, Cai et. al. [16] summarized all available ADRs into a hierarchical graph by organizing them from specific to generic categories. Therefore, a pharmaceutical company may be interested in finding the substructures that are responsible for a particular group of ADRs, which will provide an early guideline for avoiding those related substructures or their continuous spectrum of representations [17]. To this end, we aim to group all the significant substructures-ADR relations based on guilt by association principle. In particular, we represent all such significant substructure-ADR pairs in a bipartite graph, where substructures are represented in one layer and ADRs in another layer and edge between them represents a significant association obtained from the previous step. consequently, we apply biclustering algorithms [18] to find the higher level groupings (bi-cliques) of substructure-ADR pairs.

### Evaluation

In this section, we will describe the evaluation criteria for our proposed neural fingerprint method.

We evaluated our neural fingerprint method based on meaningful chemical features (often termed as fingerprints) from drugs that can be extracted in many different ways. Ten popular chemical fingerprints were used in our ADR prediction tasks: Shortest-path, PubChem, MACCS, CDK Standard, CDK Graph, Klekota-Roth (KR), E-State, CDK Hybridization, CDK Extended, ECFP6 (circular fingerprints). The detailed descriptions about these fingerprints are available in [19]. Fingerprints contain information about certain chemical properties of each molecule, such as the number of specific atoms, substructures, atom pairs.

Among the ten fingerprints, the circular fingerprints [14] are a recent development by extending Morgan algorithms [20], which was originally designed for the graph alignment problem to resolve molecular isomorphism. Although circular fingerprints are similar to neural fingerprints in nature, the circular fingerprint requires large number of pre-defined features and they are not specific to ADR. We used a R package [21] for extracting all ten different chemical fingerprints as mentioned above. We generated different chemical fingerprints for each molecule with default parameters, except that the maximal radius parameter was set to 4 for both circular fingerprints and the neural fingerprint method.

We evaluate the performance of our model based on two criteria: prediction accuracy and evidences from literatures about the substructure-ADR associations.

Since we built one predictive model for each ADR separately, in order to compare the performance of that predictive model across all ADRs, we used three different methods to evaluate the performance: global, row-wise(drug-wise), and column-wise(ADR-wise). For *N* drugs and *M* ADRs (endpoints) we have two matrices, an *N*×*M* matrix of original binary labels and an *N*×*M* matrix of prediction values. The global evaluation compares all the *N*×*M* original labels versus all the *N*×*M* prediction values in one time, while the row-wise and column-wise evaluations compare the original labels versus the prediction values by row (drug) and by column (ADR), respectively.

**Fig. 3:**
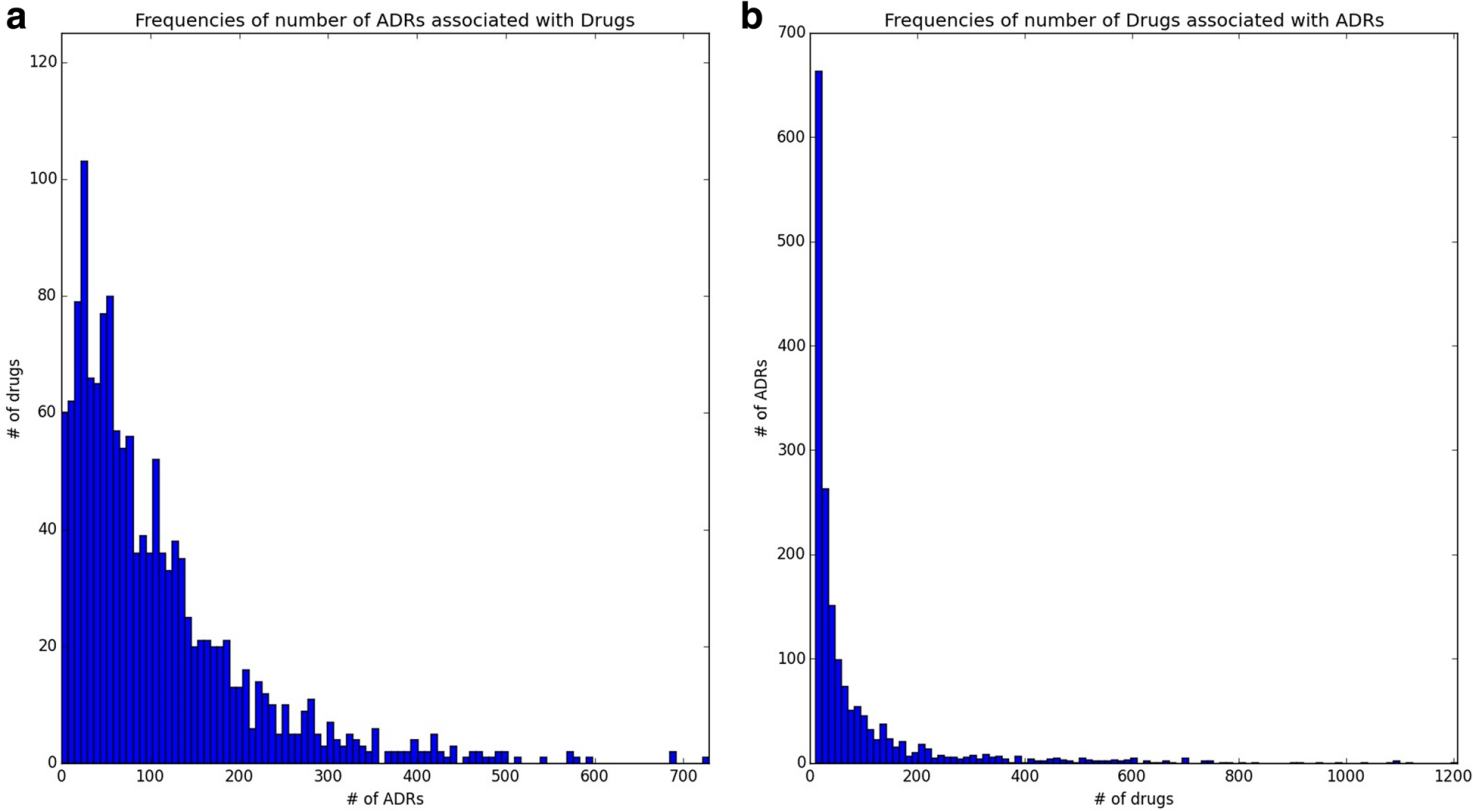
The frequencies of ADRs and Drugs: (**a**) Histogram of number of positive ADRs associated with each drug with average of 106, (**b**) Histogram of number of drugs associated with each ADR with average of 86

During our evaluation, we used 10-fold cross-validation procedure to test our predictive model. We computed several standard metrics such as accuracy, precision, recall (sensitivity), specificity, F1-score, area under the ROC curve (AUC) and area under the precision-recall curve (AUPR) [22] for global and column-wise evaluations. For row-wise evaluation, since the models were developed column-wise (by ADRs) as opposed to rows (by drugs), we only evaluated metrics related to information retrieval domain such as accuracy, precision, recall (sensitivity), F1-score and additional, P@K score. The P@K score is defined as the precision computed for top *K* predicted ADRs of each drug. This measure is very popular in drug discovery domain [23, 24], since it selectively evaluates the top ranking predictions instead of everything. Typically, each drug can have a large number of ADRs predicted by the computational model (Fig. 3a), which poses challenge for the domain experts who are interested in extensive evaluation of some specific ADRs of their interests. Therefore, it will be very useful to look at the top-most ADRs first and then evaluate successive ADRs. We used P@10 based on the common practice in literature [23].

Finally, we searched for optimal values of the hyper-parameters of our models such as regularization parameter(*λ*_1_ and *λ*_2_), maximum radius for substructures (R), number of neurons in each hidden-layer, and number of fingerprints (K) with the best F1-score [22] selected by cross-validation (CVs). We used batch normalization to optimize each batch of size 100 using the ADAM algorithm [25].

For evaluating the substructures obtained from the neural fingerprint framework, we used literature validations. If a substructure is strongly associated with a specific ADR, we may be able to identify new drugs that contain the specific substructure to cause the specific ADR. By analyzing the features, we identified substructures which are positively associated with specific ADRs. In order to test their ADR prediction capability, we looked for drugs that contain such substructures but had not been reported to cause the specific ADRs in the SIDER database. We used our developed models to predict such drugs for the ADRs, and also looked for reports of those same drug-ADR associations in the medical literature.

## Results

In this section, we will first describe the data we used. Then we show results of our neural fingerprint based framework both in terms of how it improves the prediction power of ADRs and how to find meaningful substructures that are associated with the ADRs.

### Data preparation

We harvested drug-ADR associations from the Side Effect Resource (SIDER) database [26], which was generated by mining the text information from drug labels. We used SIDER version 4.1 (http://sideeffects.embl.de/) as our training set, which contains 1430 drugs and 6123 side-effects (Preferred Terms) with 166,128 unique drug-ADR associations.

We converted the STITCH IDs of drugs from SIDER into PubChem IDs [27] and downloaded their structure information from PubChem. SIDER contains both Lowest Level Terms (LLT) and Preferred Terms (PT) from MedDRA for ADRs [28]. We selected Preferred Terms for ADRs as our endpoints, because they contain the higher level summarization of multiple synonymous or verbatim lower level terms. We also analyzed the frequency of drugs associated with each ADR and it turned out that the number of drugs associated with ADRs varies a lot as shown in Fig. 3b. The ADRs with 10 or fewer drugs don’t have enough positive samples and were removed from the analysis, thus, we ended up with 1766 ADRs and 1430 drugs from SIDER for ADR prediction and 151,501 total drug-ADR associations.

### Prediction performance

**Fig. 4:**
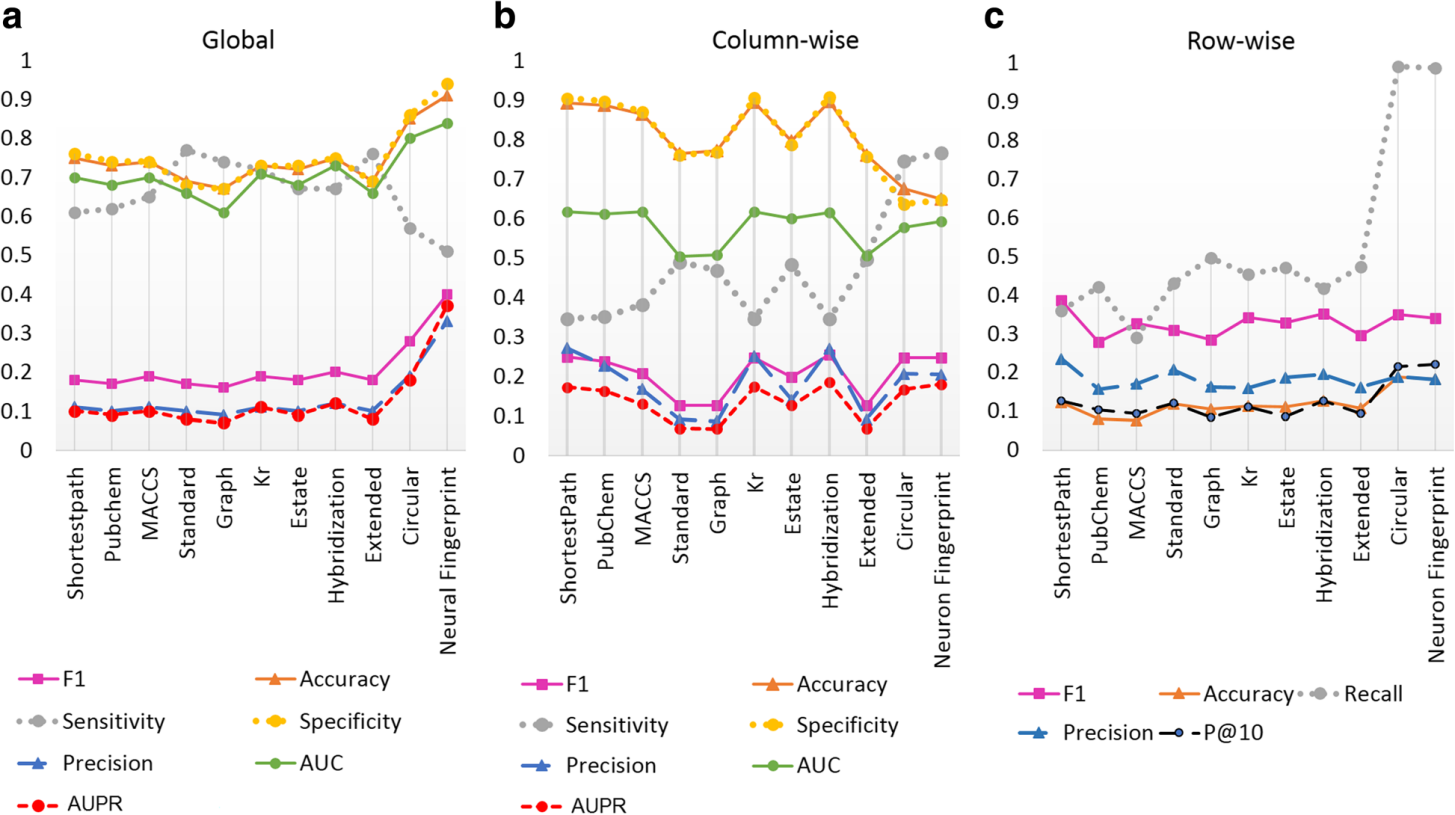
Predictive performance Comparison of different fingerprint methods on SIDER dataset based on (**a**) global, (**b**) column-wise (by ADR) and (**c**) row-wise (by drug) evaluations

We summarized the prediction performance of eleven different fingerprint algorithms including neural fingerprint on the SIDER dataset in Fig. 4. Figure 4a represents the global evaluation of our prediction models on SIDER with the representation of 50 fingerprints in the final layer for neural fingerprints and an optimum value of sparsity threshold *λ*_1_=0.0001 and *λ*_2_=0.01 and one hidden layer with 100 neurons for the final level neural network built on top of the fingerprints. We can clearly observe that the circular fingerprints and neural fingerprints performed the best among all different methods in terms of all evaluation metrics except sensitivity. In particular, the neural fingerprints had the best F1 score and AUC in global evaluation criteria, while neural fingerprints, circular fingerprints and hybridization fingerprints performed the best when evaluated column-wise (by ADR) as in Fig. 4b. In the row-wise evaluation metrics in Fig. 4c, neural fingerprints performed the best in terms of P@10 (precision at top 10 predictions, where K = 10) with a reasonable F1 score.

Table 2 listed the top 10 ADR prediction models using neural fingerprints ranked by AUC. We found that our models performed well on a variety of ADRs in terms of AUC including (1) skin-related ADRs such as dermatitis perioral, skin striae and acneiform eruption, (2) metabolic-related ADRs including alkalosis hypokalaemic and increased insulin requirement, (3) muscle-related steroid myopathy and (4) eye related cataract subcapsular. However, it could be possible that the drugs under these categories of ADRs have some common structural properties so that they are easier to be differentiated by structure-based models.

**Table 2:** Top 10 ADR models ranked by AUC

ADR concept ID	ADR name	Number of positive drugs	Accuracy	Sensitivity	Specificity	Precision	AUC
C0263449	Dermatitis perioral	31	0.945	0.742	0.950	0.247	0.957
C0085570	Alkalosis hypokalaemic	15	0.910	0.867	0.911	0.094	0.935
C0270994	Steroid myopathy	16	0.892	1.000	0.890	0.094	0.931
C0235409	Increased insulin requirement	15	0.852	1.000	0.850	0.066	0.927
C0085660	Aseptic necrosis	18	0.908	0.778	0.910	0.099	0.916
C0877365	Infusion site erythema	11	0.936	0.545	0.939	0.065	0.913
C0271738	Secondary adrenocortical insufficiency	25	0.884	0.880	0.884	0.119	0.908
C0175167	Acneiform eruption	35	0.945	0.714	0.951	0.266	0.905
C0235259	Cataract subcapsular	35	0.913	0.800	0.915	0.192	0.905
C0152459	Skin striae	52	0.977	0.788	0.984	0.651	0.902

The model evaluation metrics during cross-validations are provided

**Fig. 5:**
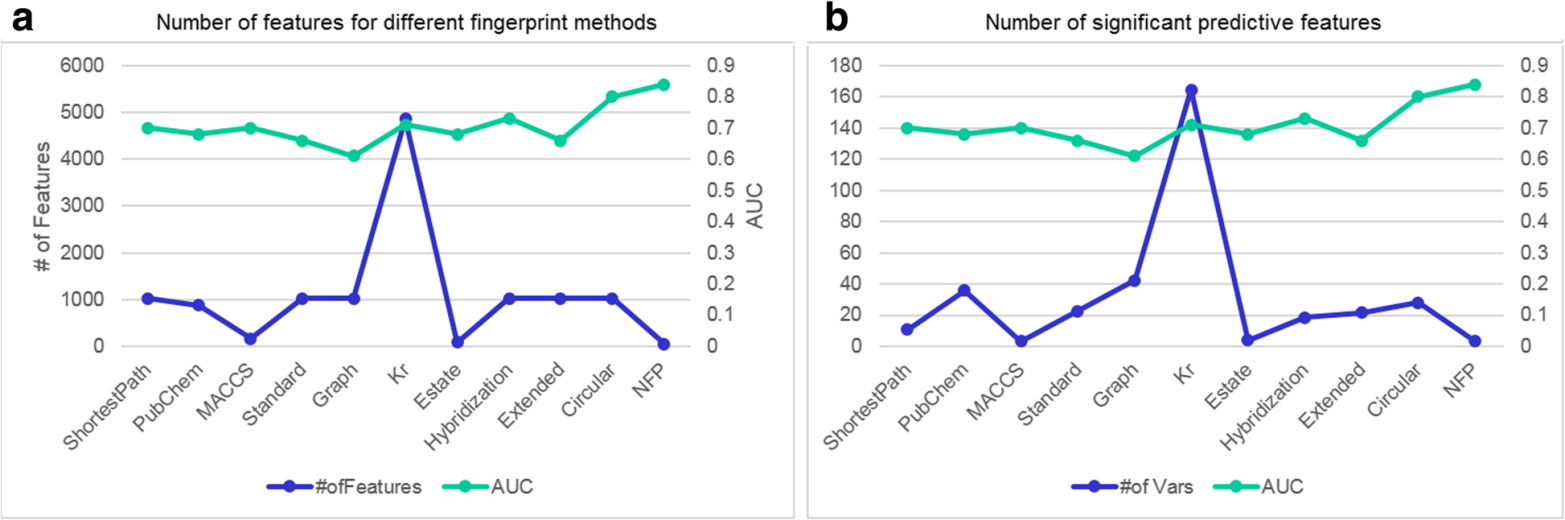
Number of features used and selected by different methods Panel (**a**) shows the average number of features defined by different chemical fingerprint methods in left y-axis and area under the curve (AUC) in right y-axis. Panel (**b**) shows the average number of significant features that are predictive of ADRs in left y-axis and AUC in the right y-axis. The proposed neural fingerprint (NFP) have better predictive power than other fingerprints, although it uses significantly less number of features than other techniques

We also analyzed the relationship between the numbers of features and prediction performance and included the results in Fig. 5. The neural fingerprint method used the least numbers of features (50 for this study) than other methods (1024 for most of them) and achieved much better performance. Some other methods such as MACCS and E-State also generate a small number of fingerprints. It seems that a larger number of fingerprint features do not necessarily guarantee a better performance in this particular study of ADR prediction. Based on these results, we believe the neural fingerprint algorithm has an overall good performance and would like to use its results for feature analysis. In the following section, we will describe two case studies of the associations between chemical substructures and ADRs from the neural fingerprint results.

### Case study 1: ADR prediction for aseptic necrosis

**Fig. 6:**
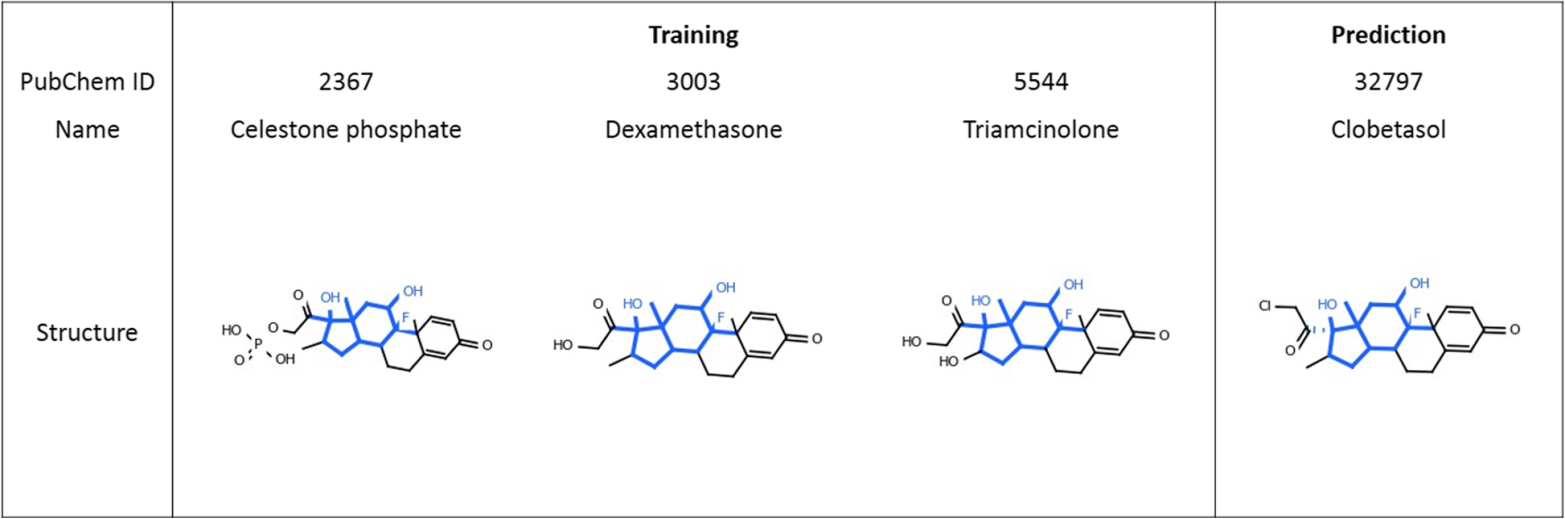
Case Study 1: Drugs structures for the training and prediction of aseptic necrosis (UMLS ID: C0085660). The highlighted substructures within the chemical structures were identified as important features for predicting this ADR

The model based on neural fingerprints obtained an F1-score of 0.176 and an AUC of 0.916 for predicting aseptic necrosis (C0085660) as an ADR. Through feature analysis, we identified the top substructures that contributed to the prediction and highlighted one of them in Fig. 6. We construct the confusion matrix for this substructure as shown in Table 3 to perform statistical analysis. It turned out that this substructure is significantly associated with aseptic necrosis with a significant chi-square test *p*-value of 1.20×10^−22^ (less than 0.0001) and odds ratio of 141. Our model predicted 5 drugs with this particular substructure that are associated with asceptic necrosis, which served as important features for the model to predict all of them to cause the ADR of aseptic necrosis. Three out of these five drugs were labeled in SIDER dataset to cause this ADR, which are shown in the left panel of Fig. 6 with this particular substructure highlighted in blue. More importantly, our model successfully identified the fourth compound, Clobetasol, as a cause of this ADR. We looked up the literature and found D. J. Hogan et al. [29] reported a case study that long-term use of Clobetasol propionate led to aseptic necrosis of the hips; therefore, our prediction is validated and we believe the substructure in Fig. 6 have a positive association with aseptic necrosis.

**Table 3:** The confusion matrix to evaluate the association between substructure in Fig. 6 and ADR aseptic necrosis

	Drugs have substructure	Drugs dont have substructure
Aseptic necrosis	3	15
No aseptic necrosis	2	1410

### Case study 2: ADR prediction for back pain

**Fig. 7:**
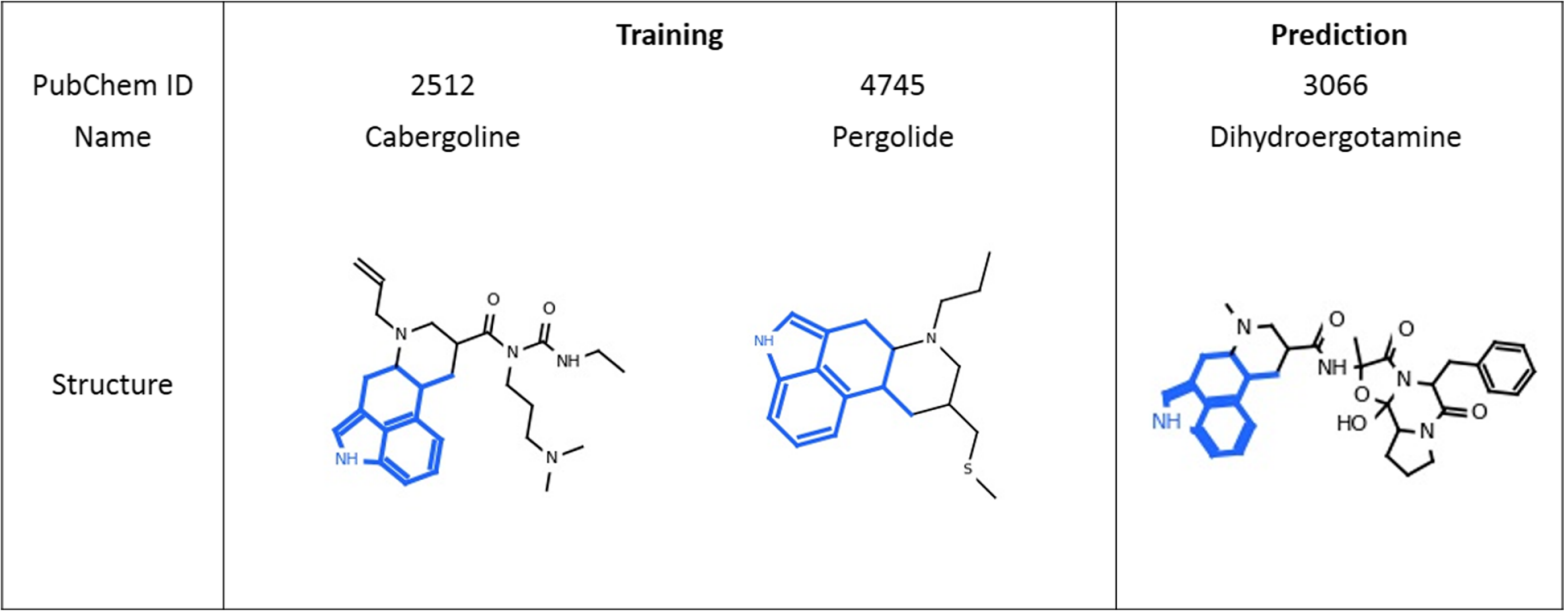
Case Study 2: Drugs structures for the training and prediction of back pain (UMLS ID: C0004604). The highlighted substructures within the chemical structures were identified as important features for predicting this ADR

Likewise, we examined our model prediction on back pain (C0004604). Pain-related ADRs are usually very hard to predict, and Wang et al. [30] predicts those with AUCs 0.62 in average even by combining chemical structure with transcriptome data. Our method, by using only chemical structure information, obtained an F1-score of 0.520 and an AUC of 0.590. One of the substructures that contributed to the prediction is highlighted in Fig. 7. Though the association between this substructure and back pain is not statistically significant (*p*≥0.05) from the SIDER database, the odds ratio is 3.71 (larger than 1), indicating a positive effect. From Fig. 7, we see that two compounds with this substructure, AC1L1DUH and AC1L1IV2, were labeled to cause this ADR in SIDER. The third compound, Dihydroergotamine, also contains the same substructure but wasn’t labeled to cause back pain in SIDER. However, our model successfully predicted this compound to cause back pain. This was also reported on drugs.com (https://www.drugs.com/sfx/dihydroergotamine-side-effects.html). These three molecules are relatively diverse in other parts of their structures; however, the highlighted substructure is the major identical part across them which was given an important weight by our model. From the results of our feature analysis, it is possible that this substructure structure is associated with back pain.

## Discussion

In this section, we discuss the grouping of obtained significant substructures and link our ADR prediction methods with drug safety signal detection.

### Higher-level grouping of the obtained substructures

**Fig. 8:**
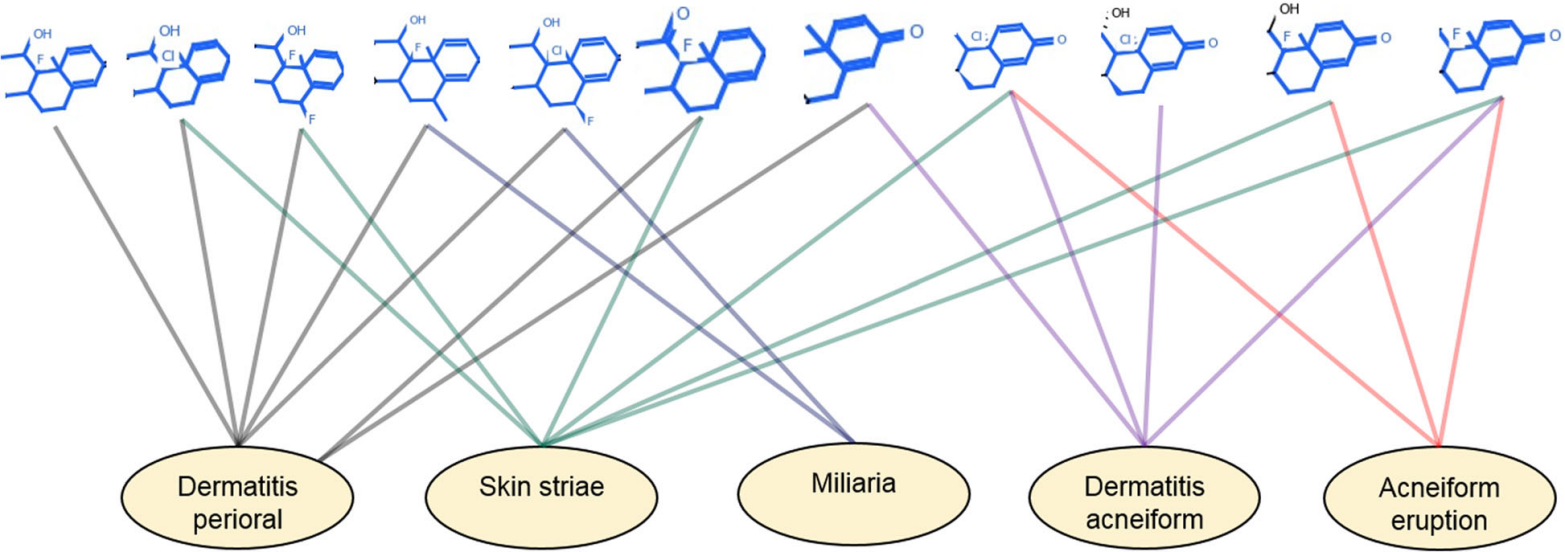
Higher Level representation of significant substructure-ADR associations: Our biclustering algorithms on the bipartite graph containing significant substructures discovered a family of similar chemical structures from Cortisols that are mostly that are associated with many of skin related ADRs

We also represented the significant substructure-ADR associations into a bipartite graph and then perform a biclustering algorithm proposed by Cheng and Church [31] due to its flexibility to find noise-tolerant coherent bi-clusters. Figure 8 shows the largest bi-cluster containing the a group of substructures and ADRs. Each edge in this graph represents a significant substructure-ADR associations below *p*-value ≤0.05. Interestingly, all of the ADRs belong to either skin or other related ADRs. On the other hand, all the significant structures that are associated with these ADRs are minor structural variations of each others, often with a change of Halogen atom while binding with the Benzine group, which may be useful for inferring useful domain knowledge.

From the above examples, we believe our models not only have the capability to predict ADRs, but also could identify the substructures that potentially play an important role in causing a specific or a group of ADRs. After the identification of important substructures, additional statistical analysis can provide mathematical assessments of such associations. We believe the structure-based machine learning model combined with feature extraction, substructure identification, statistical and bi-clustering analysis provide a systematic evaluation of the associations between chemical structures and ADRs. It may not only help the researchers to study the structural triggers and provide clues for underlying mechanisms of ADRs, but it may also guide the drug developers to modify the suspicious substructures to possibly prevent the ADRs from happening.

### Complementary approach to drug safety signal detection

Our method uses only chemical fingerprints to predict ADRs which is often available in pre-clinical stages. Therefore, it can be used as a complementary approach to post-marketing drug surveillance models, which is built on the case reports to drug administration agencies (US FDA’s Adverse Event Reporting System (FAERS) [32]). In order to further characterize our method against these models, we conducted additional experiments on four popular ADRs (Acute Kidney Injury, Acute Liver Injury, Acute Myocardial Infarction and GI Bleeding) which usually are studied in the scenarios of safety signal detection. We adopted OMOP dataset [33] as the gold standard, and curated 172 drugs in total which have associations with these four ADRs, similar to Xiao et al. [34]. OMOP dataset provides both positive and negative drug-ADR associations which are well-validated by domain experts in contrast to SIDER dataset which provides only the positive associations (the negative associations were assumed for any missing drug-ADR associations).

**Fig. 9:**
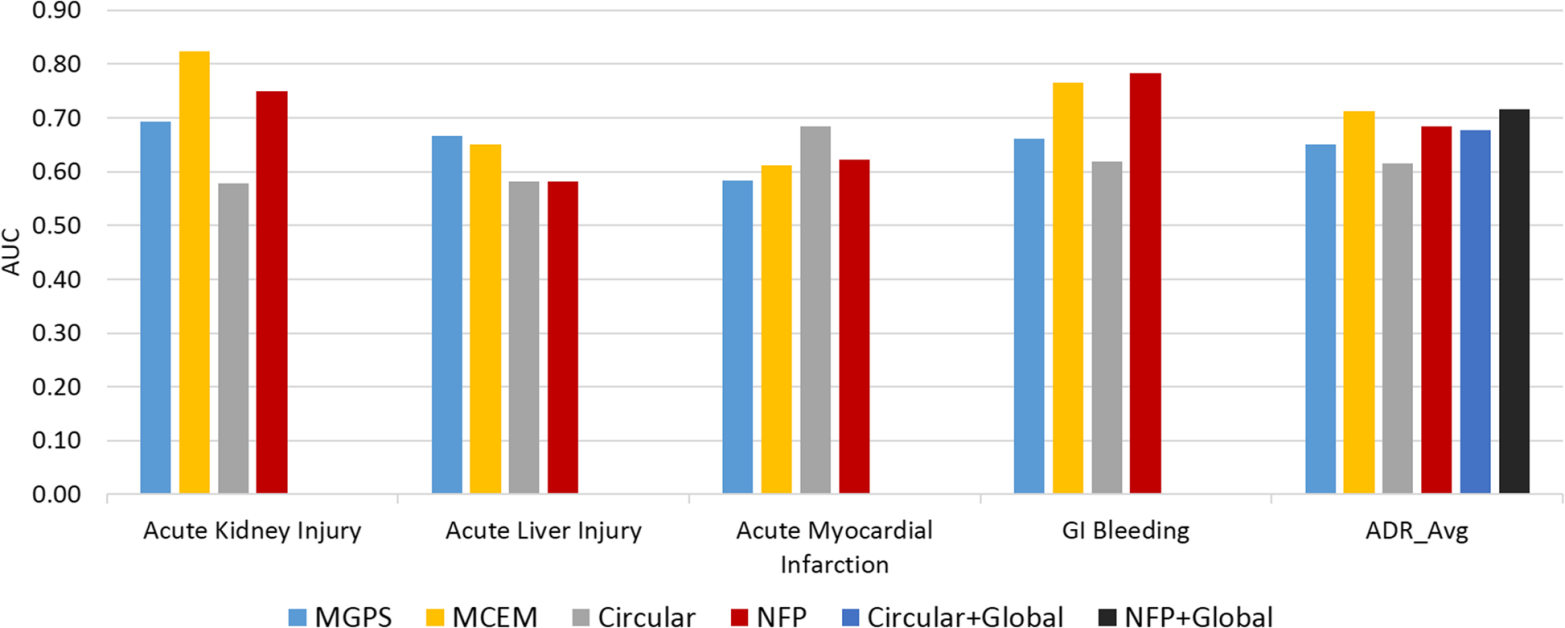
AUC comparisons of NFP method with three models on OMOP benchmark dataset: OMOP data consists of gold standards of four ADRs: Acute Kidney Injury, Acute Liver Injury, Acute Myocardial Infarction, and GI Bleeding. The other ADR prediction model is Circular fingerprint method. Signal detection methods include MGPS and MCEM, and results come from [34]

We compared our NFP method with Circular fingerprint method on the OMOP benchmark dataset. In addition, we compared two drug safety signal detection algorithms: Multi-item Gamma Poisson Shrinker (MGPS) [35], and Monte-Carlo Expectation Maximization (MCEM) [34]. The AUC comparisons on four ADRs (Acute Kidney Injury, Acute Liver Injury, Acute Myocardial Infarction, and GI Bleeding) and their averages (ADR-wise evaluation) are summarized in Fig. 9. In addition, the global evaluation results are also shown for the two chemical fingerprint methods: NFP+Global and Circular+Global (the drug-wise evaluation is not feasible since the number of ADRs are only four here). Our NFP method significantly outperforms Circular fingerprint method on three ADRs: Acute Kidney Injury, Acute Liver Injury, and GI Bleeding, and on overall ADR-wise averages.

Moreover, in Fig. 9, even comparing to MGPS and MCEM algorithms, our NFP model provided similar performances in terms of AUC. Even, the global NFP model (AUC = 0.72) slightly outperforms the average of best signal detection model, MCEM (AUC = 0.71). Note that we used only chemical structures data which are not as rich as the FAERS case reports which contain direct information about adverse event observations, but still can achieve reasonable performances in very early stage of drug design. This further demonstrates that our method provides a complementary way of ADR prediction to drug safety signal detection.

## Related work

Existing studies for ADR prediction utilized diverse data sources, such as biological pathways [36], chemical-protein interactions [37], and post-market surveillance data, to predict ADRs [38]. However, many of these data types are based on either experimental results or post-market reports which take time and money to generate or harvest [39]. In order to predict ADRs for a drug candidate in an early stage of drug development, predictions need to be made using limited available information such as chemical structures [4, 7]. The existing structure-based approaches can be summarized into two categories, similarity-based approaches and machine learning-based approaches.

The similarity-based approaches predict ADRs by looking for molecules that are structurally similar to the existing drugs [23, 40, 41]. Though they are relatively simple to implement, these methods are less effective if the existing and predicted drugs are diverse in structure. Also, they treat all the structural features with equal weight and do not optimize for each specific ADR. Moreover, these models are harder to interpret for finding chemical structures responsible for ADRs.

The machine learning-based approaches utilize the molecular fingerprints [19] such as PubChem fingerprints [42] and the circular fingerprint [14] to build up models for ADR prediction using various types of models, such as Bayesian network [43], decision tree [44], and canonical correlation analysis based approaches [4]. However, most of the existing machine learning-based approaches define the fingerprints *a-priori* from domain knowledge [4, 6–8] and thus are not able to explore all possible chemical substructures. Moreover, they separated the fingerprint generation and the model development phases into two separate steps. Recently, a deep learning method emerged to learn a concise set of fingerprints automatically from the given set of drugs without using any prior knowledge [9]. However, such method was developed for predicting drug solubility and has not been applied for ADR prediction. In addition, very few studies aimed to interpret [8, 43] the obtained models, although these studies were interested in only one [8] or a very few pre-selected ADRs [43]. Therefore, how to systematically extract meaningful chemical substructures from the obtained figherprints, how to evaluate their associations with ADRs and how to summarized them into higher level groupings are not well explored, which are the focuses of our study.

## Conclusion

In this paper, we harvested drug-ADR associations from the SIDER database, and generated ten different types of chemical fingerprints from molecular structures. We developed L2 norm regularized logistic regression models for all fingerprints to predict ADRs, and also leveraged a convolutional deep learning framework to integrate neural fingerprint generation and model development. We evaluated the performance of all eleven models and found that the neural fingerprints achieved the best overall performance. Based on the outputs from the neural fingerprints, we extracted the chemical substructures of the drugs that might be associated with specific ADRs, evaluated their associations using statistical analysis and found evidence in two case studies. The proposed structure-based models can not only obtain good performance in ADR prediction, but also identify the potential connections between substructures and ADRs. This study provides a useful workflow for drug developers to identify risky substructures and may potentially help to improve the safety evaluation of pipeline drugs.

This study can be extended in multiple directions in the future in terms of both features and models. Sometime, the severity of a particular ADR is available in the SIDER dataset, which can be taken into account during model development. At the same time, since ADRs have some hierarchical structures, it is possible to develop some hierarchical classifiers to improve prediction performance. Furthermore, other types of available data sources such as chemical-protein binding and gene expression data can be integrated into our models for a data-driven approach for ADR prediction.

## Abbreviations

ADRs: Adverse drug reactions
AUC: Area under the ROC curve
AUPR: Area under the precision-recall curve
CVs: Cross-validations
FAERS: FDA’s adverse event reporting system
LLT: Lowest level terms
MCEM: Monte-Carlo expectation maximization MGPS: Multi-item gamma poisson shrinker
NFP: Neural fingerprint
PT: Preferred terms
WHO: World health organization
